# Why do adult women in Vietnam take iron tablets?

**DOI:** 10.1186/1471-2458-6-144

**Published:** 2006-06-06

**Authors:** Ritsuko Aikawa, Masamine Jimba, Khan C Nguen, Yun Zhao, Colin W Binns, Mi Kyung Lee

**Affiliations:** 1 Institute for International Cooperation, Japan International Cooperation Agency, Tokyo, Japan; 2 Department of International Community Health, Graduate School of Medicine, University of Tokyo, Tokyo, Japan; 3 National Institute of Nutrition, Hanoi, Vietnam; 4 School of Public Health, Curtin University of Technology, Perth, Australia; 5 Division of Health Sciences, Murdoch University, Perth, Australia

## Abstract

**Background:**

Conducting iron supplementation programs has been a major strategy to reduce iron deficiency anemia in pregnancy. However, only a few countries have reported improvements in the anemia rate at a national level. The strategies used for control of nutrition problems need regular review to maintain and improve their effectiveness. The objective of this study was to analyze the factors in compliance with taking iron tablets, where daily doses of iron (60 mg) and folic acid (400 μg) were distributed in rural Vietnamese communes.

**Methods:**

A cross sectional survey was conducted in Nghe An province, Vietnam in January, 2003. The study population was adult women aged less than 35 years who delivered babies between August 1^st ^2001 and December 1^st ^2002 (n = 205), of which 159 took part in the study. Data for the study were collected from a series of workshops with community leaders, focus group discussions with community members and a questionnaire survey.

**Results:**

Improvements in the rate of anemia was not given a high priority as one of the commune's needs, but the participants still made efforts to continue taking iron tablets. Two major factors motivated the participants to continue taking iron tablets; their experience of fewer spells of dizziness (50%), and their concern for the health of their newborn baby (54%). When examining the reasons for taking iron tablets for at least 5–9 months, the most important factor was identified as 'a frequent supply of iron tablets' (OR = 11.93, 95% CI: 4.33–32.85).

**Conclusion:**

The study found that multiple poor environmental risk factors discouraged women from taking iron tablets continuously. The availability (frequent supply) of iron tablets was the most effective way to help adult women to continue taking iron tablets.

## Background

Iron deficiency anemia (IDA) is one of the most frequently observed nutritional deficiencies among pregnant women in developing countries [1]. The World Health Organization estimates that the worldwide prevalence of IDA among pregnant women is 55.8% [2]. IDA is also an important risk factor in maternal morbidity [3] and results in decreased work capacity [4].

To control IDA, four approaches have been undertaken: dietary intervention, iron fortification, iron supplementation and control of parasites such as malaria and hookworm [5]. Iron supplementation is an important intervention for the IDA high-risk groups such as pregnant women [6]. To reduce IDA, at least 49 countries have implemented iron supplementation programs [7], but only a few countries have reported significant improvement in anemia rates at a national level [8,9]. This could be due to several constraints such as poor health service infrastructure [7], lack of motivation of health workers, and incomplete compliance due to side effects [5] and lack of awareness of IDA among pregnant women [10].

In Vietnam, IDA is a serious public health issue for pregnant women with a prevalence of 53% in 1995. The national iron supplementation program, economic growth and improved food availability have reduced the prevalence of IDA, but about one third are still anemic (32%) [11]. A joint government-donor-NGO working group concluded that the main cause of IDA is a lack of dietary iron intake, as Vietnam's staple food is rice and rice products [12]. A further study showed that low household consumption of meat and the presence of hookworm infection are significantly associated with a high rate of anemia [13]. In Vietnam, the communes usually have a number of health workers, including doctors, midwives and nurses in their health centers, who need to be involved in anemia programs. Vietnam has fully integrated Chinese traditional medicine into their health care system. Chinese traditional medicine refers to health practices, approaches, knowledge and beliefs incorporating plant, animal and mineral based medicines, spiritual therapies, manual techniques and exercises, applied singularly or in combination to treat, diagnose and prevent illnesses or maintain well-being [14].

In Vietnam iron supplementation programs have been a major strategy to reduce IDA in pregnancy and the dynamic nature of nutrition problems mean that strategies need regular review to maintain and improve their effectiveness. The objective of this study was to study the implementation of an iron supplementation program in two communes in rural Vietnam.

## Methods

This study was conducted in January 2003 in Yen Thanh district of Nghe An province in Vietnam. The Vietnamese Ministry of Health has conducted an iron supplementation program in 10 of the 37 communes in the Yen Thanh district since 1998. In this program, iron (60 mg) and folic acid (400 μg) tablets for daily use have been distributed to pregnant women. From the 10 communes, we purposely selected Vinh Thanh commune from the 2 communes located in low-lying areas and Quang Thanh commune from the 8 communes in higher areas. Both of these were ranked among the poorest of the communes by the district health officers of Yen Thanh district.

Data for the study were collected from a series of workshops and focus group discussions and a questionnaire survey. The commune needs were assessed during workshops held in Quang Thanh and Vinh Thanh communes. Seven commune leaders attended each workshop. They were the head of the commune, the secretary of the communist party, the head of the cultural department of the people's committee and four leaders from the Farmers Union, the Women's Union, the Youth Union, and the Retired Soldiers Union. Each two-hour workshop allowed the participants to identify the specific commune needs by assessing their importance and changeability using a participatory method. Each participant used ten seeds to score the needs items by importance and changeability. Based on their total scores of the needs items were divided into four groups by the participants: more important/more changeable, more important/less changeable, less important/more changeable and less important/less changeable. More changeable items were defined as ones that they could easily change by themselves. Less changeable were the items that they needed assistance from outside. More important items were ones that they identified to be important to obtain. Less important were the items that they identified not necessarily to obtain.

The sample for the questionnaire survey was selected from the birth registrations at the health centers of Quang Thanh and Vinh Thanh communes. Since the percentage of pregnant women who deliver at health centers is high (74%) in the north central region of Vietnam, the sample is representative of the total population [15].

The questionnaires used in the survey were modified from a similar study carried out in Nepal by UNICEF in 2001(UNICEF Nepal, unpublished). A letter of invitation was sent to all women (n = 205) aged less than 35 years old who delivered babies between August 1st 2001 and December 1st 2002 and who lived in either Quang Thanh or Vinh Thanh commune. A total of 159 women (78%) gave their consent and took part in the survey. In order to calculate the required sample size, the percentage of participants who took iron tablets for 5–9 months was estimated to be 52.3% and the relative increase percentage used in the calculations was to be 20%. On the basis of this assumption, we estimated that a sample of 188 participants was required (80% power, p < 0.05). To allow for dropouts in the study, all of the pregnant women aged less than 35 year old in two communes (n = 205) were included.

Four focus group discussions were held with participants selected among the participants of the survey in four hamlets out of 26 in Quang Thanh and Vinh Thanh communes. The focus group discussions addressed awareness of IDA, risk factors leading to women's compliance of taking iron tablets, and risk factors discouraging participants from taking iron tablets. Each focus group included a moderator and two reporters and before starting the focus group discussions, permission was sought from the participants to take notes. After the discussion, the facilitator responded to the participants' questions about health and nutrition. The reporters recorded the conversation and their own observations during the focus group discussions, which lasted 45 minutes on average. The moderator and the reporters then transcribed the conversation on the same day.

The transcripts of discussions were examined and emergent themes related to the central focus of this study were identified. The data was then categorized under appropriate headings and compared with the issues raised in the discussions. The results of the focus group discussions were then crosschecked with the questionnaire survey.

### Statistical analysis

All data of the questionnaire survey were coded and entered into SPSS version 12.0J for analysis. The women were classified into two groups, those who took iron tablets for 1–4 months (shorter groups) and the participants who took iron tablets 5–9 months (longer groups) during their pregnancy. Initially descriptive statistics were obtained and then odds ratios were calculated by using binary logistic regression analysis to evaluate the associations between duration of taking iron tablets and relevant risk factors. The risk factors included demographic factors, knowledge of anemia, health seeking behaviors and experiences of side effects. To gain some knowledge of the parasite infection risks that may affect Hb level, questions were asked about sanitation facilities. All tests were two-sided, and a significance level of 5% was regarded as significant.

This study was approved by the Ethical Committee of the National Institute of Nutrition in Vietnam. Prior to the survey, we informed all the participants that their confidentiality would be maintained. They were also told that their participation would be voluntary and that they could withdraw from this study at anytime without prejudice to their treatment. Their written informed consent was then obtained.

## Results

The socio-demographic characteristics of the sample are detailed in Table 1. There was no significant difference in the average ages of the participants, 26.4 years (SD 4.3) in Quang Thanh and 27.3 years (SD 3.6) in Vinh Thanh commune. The majority was engaged in farming and the median number of children was two. For sanitation facilities in Vinh Thanh commune, a two rooms latrine was used by 64%. On the other hand, the participants from Quang Thanh used relatively unhygienic sanitation facilities: no toilet (15%), a hole in the ground (24%), and a one room latrine (30%).

When the commune leaders ranked the commune needs in order of importance and changeability, 'improvement of IDA' was not prioritized as one of the commune needs. The items that were categorized as more important (and more changeable) were 'water safety' and 'child health' in Quang Thanh and 'roads' in Vinh Thanh commune. Other needs which commune leaders recognized as important were 'clean water', 'school building repairs', and 'health facilities' in Quang Thanh commune; 'job creation', 'drug supply', 'availability of credit to expand commune production', 'clean water', 'provision of electricity', and 'kindergarten facilities' for the Vinh Thanh commune.

The survey results showed that the program coverage was high and 97% of the participants took some iron tablets during their last pregnancy. Forty three percent of the women received iron tablets from village health workers and 20% of them from commune health workers. Few of them knew that 'taking de-worming tablets" would assist in preventing anemia (Table 3).

The percentage of those who received information on anemia from TV/radio was significantly higher in 'Longer' group (15%) than in 'Shorter' period groups (4%) (p < 0.05). The commune health workers were the main source of information about anemia (70%) compared to the women's union (31%), the village health workers (30%), or TV/radio (10%) (Table 3).

In this study, the mean duration of taking iron tablets was 4.8 months. The survey results indicated that two major factors motivated the participants to continue taking iron tablets were their experience of fewer spells of dizziness (50%), and their concern for the health of their newborn baby (54%). The focus group discussions showed that participants in all the four hamlets made efforts not to forget to take iron tablets. For example, the participants in two hamlets said, "I put iron tablets near the toothbrush", "I always place iron tablets at the same place such as under the pillow, near the tooth brush so that I do not forget to take iron tablets". The participants in one hamlet said, "I took iron tablets at the same time each evening before going to bed". Participants in three hamlets said, "I asked my husband to remind me to take iron tablets". They made other efforts to make taking the tablets more pleasant. One participant said, "I wrap the iron tablets in pumpkin leaves or eat them with other foods to disguise their bad taste and smell" (Table 2).

The survey results also showed the three major risk factors discouraging participants from starting to take iron tablets at the correct time during their pregnancy were not attending the commune health center (39.6%), iron tablets not being distributed (35.8%), and not being aware that they were pregnant (11.3%). The focus group discussions in all the four hamlets showed that the participants relied on the seasonally available food. They mainly consumed rice, seasonal available vegetables and fruits, and small portions of fish and meat (Table 2).

The risk factors that may influence duration of taking iron tablets were compared between the 'Shorter' and 'Longer' period groups (Table 3). The average duration of taking iron tablets was significantly longer in women who received information from radio/TV (p < 0.01), had a frequent supply of iron tablets (p < 0.001) and who did not experience side effects (p < 0.005). The odds ratios for a longer period of taking iron tablets (5–9 months) were significantly associated with receiving 'information from radio/TV' (OR = 22.02, 95% CI: 2.70–179.77), 'frequent supply of iron tablets' (OR = 11.93, 95% CI: 4.33–32.85), 'having a healthy new born being a reason for taking iron' (OR = 2.69, 95% CI: 1.02–7.10), 'not having side effects' (OR = 2.72, 95% CI:1.01–7.30), 'not being a farmer' (OR = 5.51, 95% CI:1.08–28.15), and 'knowing that lack of iron rich foods is a cause of anemia' (OR = 0.21, 95% CI:0.07–0.60) (Table 4). The significant factors where then incorporated into a multiple regression equation using a backward elimination procedure with similar results (Table 5).

## Discussion

This study found that multiple poor environmental risk factors and low motivation discouraged women from taking iron tablets continuously. The low priority of IDA prevention by commune leaders was often identified as one of the environmental risk factors of taking iron tablets. The results of the workshops showed that the commune leaders prioritized 'water safety', 'child health' and 'roads' above 'IDA prevention during pregnancy'. In Vietnam, commune leaders allocate the budget for commune health workers and for prevention programs. Therefore, advocacy to commune leaders is urgently needed to motivate commune health workers and to provide them with resources. One of the main problems identified in past large-scale programs for pregnant women was the lack of motivation of frontline health workers [5]. A previous study in Vietnam indicated that the availability of professionally educated nutritionists was a factor in the success of nutrition improvement projects [16].

The poor distribution system of iron tablets was identified as an environmental risk factor of taking iron tablets continuously. Logistic regression analysis indicated that longer duration of taking iron tablets was associated with a regular supply of iron tablets (OR = 11.93, 95% CI: 4.33–32.85) as shown in Table 4. An improved distribution system of iron tablets would lead to pregnant women's compliance. For example, a system where the village health workers distribute 30 iron tablets every month would be more effective in improving compliance when compared to the present system where commune health workers distribute 100 iron tablets at less regular antenatal check-ups. Those with a more frequent supply of iron tablets took them for longer period than those with a less frequent supply. It is reported that anemia prevalence decreased in Thailand in the 1980's when village health volunteers made more effort to encourage pregnant women to attend antenatal care services [9]. In this study we found that women received information on anemia from commune health workers rather than from village health workers. Health education conducted by commune health workers was limited as they spent most of the time providing treatment. Therefore, more effort is needed to mobilize village health workers to distribute iron tablets and information on anemia prevention.

Experiencing side effects was a risk factor for taking iron tablets for a shorter period of time. Past studies have shown the different forms of iron have reduced side effects and newer forms of iron such as sprinkles, candies, and beverages, [17-20] have the potential to reduce the side effects and thus increase the success of the treatment of IDA. Taking iron tablets for a longer period (5–9 months) was significantly associated with receiving information from radio/TV (OR = 22.02, 95% CI: 2.70–179.77). However women's knowledge about anemia was not identified as one of the factors in the longer period of taking iron tablets. One explanation may be that "getting information from radio/TV" may only be more important as a surrogate indicator of income.

Interestingly, women who knew that lack of iron rich foods was a cause of anemia took iron tablets for a shorter period than those who had more knowledge. It is hypothesized that those who relied on iron intake by eating iron rich foods did not take iron tablets continuously (Table 3). In our study, Chinese traditional medicine, which emphasizes iron intake from diet rather than an iron supplementation, was found to be a risk factors of low hemoglobin concentration [21].

The high level of motivation of a women to deliver a healthy baby was also identified as a factor that increased the duration of taking iron tablets. One of the difficulties in program management for anemia reduction was the low level of awareness of the target population [22]. Among the women awareness of the need for taking iron tablet is already high (98% took some tables), but they were not aware of the need to take the iron tablets continuously and to commence early in pregnancy. It is important to reinforce this program message to achieve maximal effect. Communication strategies need to be reviewed and adjusted to the women's own experiences and knowledge. For example, as women become used to taking iron supplements, different messages may be needed to promote long-term compliance.

The cutoff point of the duration of taking iron tablets (1–4 month and 5–9 months) was used to compare two groups of approximately similar numbers of participants and to correspond with the time period found in the literature to produce a response. In a study of the duration of iron supplementation it was found that 12 weeks (three month) of iron supplementation, (2,400 mg Fe or 40 tablets × 60 mg), was sufficient to produce a maximal hemoglobin response [23,24] where the compliance was 100%. However because in the real world compliance is never 100%, four months of iron supplementation was set as the criteria of success. In a comprehensive review of randomized control trials on prenatal iron supplementation, the optimal duration of taking iron tablets remains unclear [25].

No impact was observed by any socio-demographic characteristics on taking iron tablets for longer period in this study. Our survey results showed that 97% of the pregnant women living in the target communes had been supplied with iron tablets. Therefore, we can conclude that the health service infrastructure for delivery system of iron tablets in Vietnam is adequate. Health service infrastructure is one of the elements required for a successful iron supplementation program based on past experiences of iron supplementation programs [10].

Some limitations need to be considered when interpreting the results of the study. First, as the current survey was conducted entirely within a rural district, differences of geographic location were not assessed. Second, social services, such as education and health care, were evenly distributed throughout the study area though our target population was the poorest among the neighbour communes. The overall equality in social development and opportunity limited the comparison of our participants' social backgrounds. Third, this study shows the importance of having village health workers distribute iron tablets on a monthly rather than in larger quantities. However village health workers may not always be available in Vietnam to increase the frequency of distribution.

## Conclusion

This study has described the multifactoral IDA control program in rural Vietnam and found several areas for improvement of the national iron supplementation program for pregnant women. The implementation of these results will help in the development of nutrition education programs and improved distribution system of iron tablets. Further studies are needed to expand knowledge of practical policies to further reduce the burden of IDA in the commune.

## Abbreviations

IDA: Iron Deficiency Anemia

NGO: Non Governmental Organization

OR: Odds ratio

CI: Confidential interval

## Competing interests

The author(s) declare that they have no competing interests.

## Authors' contributions

RA is the principal investigator. MJ is co-investigator. VT is co-investigator in Vietnam. YZ performed the statistical analysis. CWB participated in the design of the study, statistical analysis and manuscript review, MKL assisted drafting the manuscript. All authors read and approved the final manuscript.

## Pre-publication history

The pre-publication history for this paper can be accessed here:



## Figures and Tables

**Table 1:** Socio-demographic characteristics of the women who participated in the survey

		Quang Thanh Communes (n = 74)	Vinh Thanh Communes (n = 85)	P value
Age (y) Mean ± SD		26.4 ± 4.3	27.3 ± 3.6	0.143
Occupation (%)	Farmer	94.6	83.1	0.026^3^
	Teacher	10.6	10.8	
	Others	7.1	6.1	
Education (%)	Did not compete second school	29.7	2.4	P < 0.001^3^
	Completed secondary school	71.3	97.6	
Number of children Mean ± SD		2.1 ± 1.3	1.9 ± 1.0	0.301
Sanitation facility (%)	No latrine	14.9	3.5	P < 0.001^3^
	Hole in the ground	24.3	0.0	
	One room latrine	29.7	30.6	
	Two rooms latrine	31.1	63.5	
	Toilet with water	0.0	2.4	

**Table 2:** Summary of focus group discussion

	Quang Thanh 1	Quang Thanh 2	Vinh Thanh 1	Vinh Thanh 2
What the village health workers do	Communicated about health care during pregnancy	Communicated about health care and suggested to take iron tablets during pregnancy	Communicated about health care, suggestion for antenatal check ups, healthy diets	Communicated about health care check up, good foods during pregnancy, work load
Experience that iron tablets improved	No headache, dizziness, and tiredness	No headache, dizziness, and tiredness	No headache, dizziness.	No headache, sleep and eat well
Experience of having any side effects	Bad smell, vomiting.	Bad smell, vomiting. black stool	Bad smell, vomiting.	Bad smell, vomiting, not good feeling black stool
What made to continue taking Iron tablets	Better feeling they had	Better health status they had	Better health status they had. Think of the benefits of the infants	Better health status for both mother and the fetus
Efforts not to forget taking iron tablets	Placed iron tablets at the same place Asked husbands to remind them	Placed iron tablets under the pillow Took iron tablets with other foods(sweat potato, pumpkin leaves)	Remembered themselves Asked husbands to remind them	Took at the same time Asked husbands to remind them
Events to stop taking iron tablets	Felt healthier Broken iron tablets given	Broken iron tablets given	Tried to take it regularly	Broken iron tablets given
Encouragement from family members	Not mentioned specifically	Not mentioned specifically	Not mentioned specifically	Not mentioned specifically
Dietary pattern	Mainly rice and more fish than meat. Available and seasonal vegetables and fruits in the region	Mainly rice and more fish than meat. Available and seasonal vegetables and fruits in the region	Mainly rice and more fish than meat. Available and seasonal vegetables and fruits in the region	Mainly rice and more fish than meat. Available and seasonal vegetables and fruits in the region

**Table 3:** Comparison of risk factors that affect duration of taking iron tablets^1 ^(%)

	Shorter Duration (n = 73)	Longer Duration (n = 79)	P (χ^2 ^analysis)
Knowledge on anemia			
Lack of iron rich foods is cause of anemia	60.3	43.0	0.036
Meat is one of the iron rich foods	53.5	46.5	0.043
Beans is one of the iron rich foods	61.3	34.2	0.001
Increased requirement is cause of anemia	37.0	25.3	0.160
Taking iron tablet is to prevent anemia	71.2	75.9	0.582
Taking de-worming tablets is to prevent anemia	1.4	0.0	0.480
Difficulty pregnancy is consequence of anemia	28.8	20.3	0.259
Small baby is consequence of anemia	57.5	48.1	0.263
Unhealthy child/mother is consequence of anemia	63.0	68.4	0.499
Advice on side-effects being told	34.2	29.1	0.601
Information source of anemia			
Commune health worker	64.4	77.2	0.107
Women's union	31.5	32.9	0.864
Village health worker	27.4	32.9	0.485
Radio/TV	4.1	15.2	0.029
Health seeking behaviors			
Western medicine preferred	75.8^5^	62.2	0.251
Frequent check ups during pregnancy (>3 times)	86.3	92.4	0.292
Dizziness stopped is a reason for taking iron tablets	51.4	49.4	0.871
Health new born is a reason for taking iron tablets	48.6^7^	63.3	0.074
Environmental risk factors			
Hygiene latrines use	57.5	43.0	0.104
Experiences of side effects	46.6	24.4^7^	0.006
Iron tablets supplied before pregnancy	54.8	72.2	0.029

Note: Seven respondents had missing data, five for not taking iron tablets and two for not responding to questions on duration of taking iron tablets.

Shorter duration means the participants took iron tablets for 1–4 months during last pregnancy.

Longer duration means the participants took iron tablets for 5–9 months during last pregnancy.

Hygiene latrines have two rooms, a latrine and a latrine with water.

**Table 4:** Odds ratios of lower and higher risk groups of taking iron tablets for longer period

		Odds ratio	95.0% C.I.		P
Radio/TV is the information source					
	Yes	22.02	2.70	179.77	0.004
	No	1.00			
Frequencies of iron tablets supply					
	1–3 times	1.00	4.33	32.85	0.000
	4–9 times	11.93			
Occupation					
	Farmer	1.00	1.08	28.15	0.040
	Others^4^	5.51			
Experience of side effects					
	Yes	1.00	1.01	7.30	0.047
	No	2.72			
Lack of iron rich foods is cause of anemia					
	Yes	0.21	0.07	0.60	0.004
	No	1.00			
Healthy new born is a reason for taking iron					
	Yes	2.69	1.02	7.10	0.046
	No	1.00			
Sanitation facility^5^					
	Hygienic	2.31	0.85	6.33	0.102
	Not hygienic	1.00			

Odds ratio is obtained from binary logistic regression coeffic, with backward elimination procedure.

Total number of the participants that remained in a final model was 120.

Factors included in the model were: Age in years, education, religion, number of children, first place of checked up, if IEC was seen in commune, reasons of taking iron tablets, if caution for taking iron tablets was told, if iron tablets were delivered before pregnancy, place of iron tablets supplied, knowing that role of iron is good for health, if side effects was explained were also entered in the first models and did not remain in the final models. Only risk factors which partial regression coefficients that were significant (p < 0.05) were included in the final models.

**Table 5:** Multiple regression analysis with factors that affect duration of taking iron tablets, partial regression coefficients shown (n = 151)

R^2^	0.21	p-value
Receiving iron tablets at commune health center (0:no, 1:yes)	0.96	P < 0.05
Healthy new born is a reason that iron tablets are helpful (0:no, 1:yes)	1.1	P < 0.01
Side effects (0:not experienced, 1: experienced)	-1.3	P < 0.01
Preferring treatment (0:traditional, 1:western)	-0.89	P < 0.05
Frequency of check up (2: twice 3: three times 4: more than three times)	1.0	P < 0.05

Factors included in the model were: Age in years, education, religion, mother's occupation, number of children, hygiene latrine, first place of checked up, knowledge of anemia, if IEC was seen in commune, reasons of taking iron tablets, caution for taking iron tablets, experiences of side effects, if iron tablets were delivered before pregnancy, place of receiving iron tablets health worker, if side effects was explained, information source of anemia were also entered in the first models and did not remain in the final models. Only risk factors which partial regression coefficients were significant (p < 0.05) were included in the final models.

R^2 ^= is the determination coefficient.

The P-value is for the correlation between duration of taking iron tablets and its affecting factors by the multiple regression analysis.
